# The Enigma of Delayed Neurotoxicity in Organophosphate Poisoning: A Case Report of Clinical Presentation With Normal MRI Findings

**DOI:** 10.7759/cureus.62877

**Published:** 2024-06-21

**Authors:** Jayksh Chhabra, Akankshi Oberoi, Neha Pandita, Vinit Banga

**Affiliations:** 1 Neurology, Park Hospital, Patiala, IND; 2 Neurology, BLK-Max Super Speciality Hospital, New Delhi, IND

**Keywords:** organophosphate induced delayed neurotoxicity, organophosphate induced polyneuropathy, myelopathy, intermediate syndrome, organophosphate pesticide poisoning

## Abstract

Organophosphates (OP) are the most widely used pesticides globally and are misused for suicides because of their easy availability. It leads to functional impairment of distal segments of sensory and motor axons of peripheral nerves, as well as impacting the ascending and descending spinal tracts. It progresses through latent, progressive, static, and improvement phases. In the improvement phase, peripheral nerve regeneration occurs, revealing the spinal cord lesion with myelopathic features. The acute symptoms and treatments of OP poisoning have been extensively documented in the literature. Delayed neurotoxicity is a rare but debilitating condition that can manifest weeks after initial exposure. A high index of suspicion for OP-induced delayed neurotoxicity should be maintained in patients presenting with delayed neurological symptoms post-OP exposure, even with normal MRI findings. OP linked to delayed neuropathy include triorthocresyl phosphate, chlorpyriphos, malathion, fipronil, mipafox, matriphonate, and parathion. Among these, the most hazardous OP ester is tri-o-cresyl phosphate. We report a case of a 28-year-old male who developed neurotoxicity five weeks following OP poisoning with chlorpyrifos. Early diagnosis and symptomatic management are important for improving patient outcomes.

## Introduction

Organophosphates (OP) are a class of chemicals commonly used as pesticides and nerve agents, known for their potent neurotoxicity. People in middle- and low-income countries like India experience significant health effects as a result of poisoning from substances like OP [1]. Three distinct stages describe OP poisoning: acute cholinergic crisis, intermediate syndrome, and OP-induced delayed neurotoxicity (OPIDN) [2]. The intermediate syndrome manifests 24-96 hours after intake and is characterized by paralysis of the neck flexors, respiratory muscles, and limb muscles [3]. OPIDN usually takes more than seven days after the OP substance intake to present clinically [4]. While the acute effects of OP poisoning are well documented and often managed with atropine and pralidoxime, less attention is given to the chronic and delayed neurological sequelae. OPIDN is a distal axonopathy affecting both the central and peripheral nervous systems. In the peripheral nervous system, it manifests as motor-sensory polyneuropathy; in the central nervous system, it presents with myelopathic symptoms [5]. We report a case of a 28-year-old male patient who ingested an OP substance and subsequently developed delayed myelopathy, despite having normal imaging results.

## Case presentation

A 28-year-old man presented to the emergency room with altered sensorium, hyperlacrimation, hypersalivation, bradycardia, and miosis two hours after suicidal ingestion of approximately 80 ml of OP (chlorpyrifos). He was treated with gastric lavage, atropine, and pralidoxime. After two days, he had intermediate syndrome, characterized by weakness in neck muscles, difficulty holding his head up, and breathing difficulty. He was mechanically ventilated for one week. The patient gradually improved and was discharged after 25 days without any neurological deficits.

**Figure 1:**
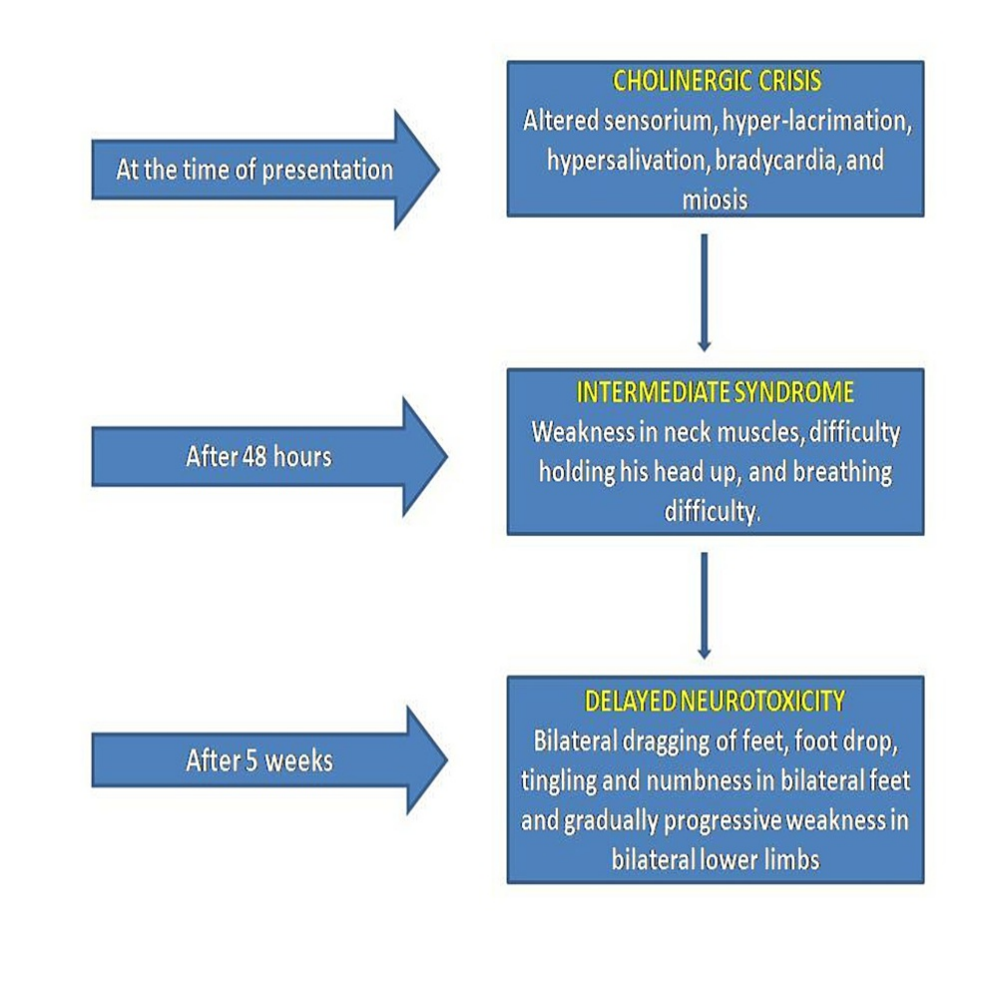
Timeline of onset of symptoms after OP ingestion Image credits: Akankshi Oberoi and Jayksh Chhabra

Five weeks later, he presented with a complaint of weakness in bilateral lower limbs, and he started having difficulty walking. He dragged his feet while walking, leading to an abnormal gait. Figure 1 illustrates the timeline of the symptom onset of different stages of OP poisoning in the patient.

The patient was oriented to time, place, and person. Deep tendon reflexes were hypoactive in bilateral lower limbs. The power of the Medical Research Council (MRC) was 5/5 in both upper limbs, 4/5 in bilateral hips and knees, and 2/5 in bilateral ankles. The patient had a high steppage gait due to a bilateral foot drop. Sensory loss of pain, touch, temperature, and joint positioning in bilateral lower limbs were present up to mid-shin. The electrodiagnosis study revealed the presence of polyneuropathy, with sensory-motor axonal involvement in both lower limbs and motor axonal involvement in the right median nerve. Higher mental functions and cranial nerve examinations were normal. The patient was put on symptomatic management with gabapentin and nortriptyline, physiotherapy, and a foot splint. On a follow-up visit after one month, the patient noticed urinary urgency with occasional incontinence. On examination, there was spasticity in bilateral lower limbs. The MRC has a power of 4/5 in bilateral hips and 2/5 in bilateral knees and ankles. Lower limb reflexes were hyperactive, and bilateral plantar responses were extensor. Sensory loss had resolved. The tone, power, and reflexes of both the upper limbs were normal. All the examinations are summarized in Table 1.

**Table 1:** Summary of examinations Power grading MRC grading MRC, Medical Research Council Table credits: Jayksh Chhabra and Akankshi Oberoi

Tone	
Right lower limb	Hypertonic
Left lower limb	Hypertonic
Right upper limb	Normal tone
Left upper limb	Normal tone
Power	
Joint power	MRC grading
Right hip extensor and flexor	4/5
Left hip extensor and flexor	4/5
Right knee extensor and flexor	2/5
Left knee extensor and flexor	2/5
Right dorsiflexion	2/5
Left dorsiflexion	2/5
Right wrist extension and flexion	5/5
Left wrist extension and flexion	5/5
Right elbow extension and flexion	5/5
Left elbow extension and flexion	5/5
Right shoulder	5/5
Left shoulder	5/5
Reflexes	
Right ankle jerk reflex	3+
Left ankle jerk reflex	3+
Right knee jerk reflex	3+
Left knee jerk reflex	3+
Right Babinski	Positive
Left Babinski	Positive
Right wrist reflex	2+
Left wrist reflex	2+
Right biceps reflex	2+
Left biceps reflex	2+
Right triceps reflex	2+
Left triceps reflex	2+

Lab investigations and CSF findings were normal, as shown in Table 2 and Table 3.

**Table 2:** Laboratory investigations ESR, erythrocyte sedimentation rate; HBV, hepatitis B virus; HCV, hepatitis C virus; SGOT, serum glutamic oxaloacetic transaminase; SGPT, serum glutamate pyruvate transaminase

Labs	Value	Reference range	Units
Hemoglobin	15	13.6-17.7	g/dL
WBC count	8.37	4-11	10*3/uL
Platelet	307	150-450	10*3/uL
Blood sugar	90	70-100	mg/dL
Blood urea	23	6-24	mg/dL
Serum creatinine	1.04	0.7-1.3	mg/dL
Sodium	144	134-146	mEq/L
Potassium	4.4	3.5-50	mEq/L
Serum ionized calcium	1.13	1.12-1.32	mEq/L
ESR	10	0-20	mm/hr
Vitamin B12	624	187-883	pg/mL
SGOT	26	5-40	U/L
SGPT	41	7-56	U/L
Serology (HIV, HBV, and HCV)	Negative		

**Table 3:** CSF analysis

Lab	Value	Reference range	Units
Glucose	65	50-76	mg/dL
Protein	48	15-50	mg/dL
RBC	Nil	0-10	cells/mm^3^
WBC	<5	0-5	cells/mm^3^

An MRI of the whole spine, focusing on the individual cervical, thoracic, and lumbosacral regions, was done, which was normal. He was put on muscle relaxants with subsequent increasing doses, multivitamins, and anxiolytics. Intensive physiotherapy and rehabilitative management for the lower limbs were also started. He follows up every three months and is doing well overall on this treatment, gradually improving his power and spasticity of the lower limbs.

## Discussion

**Figure 2:**
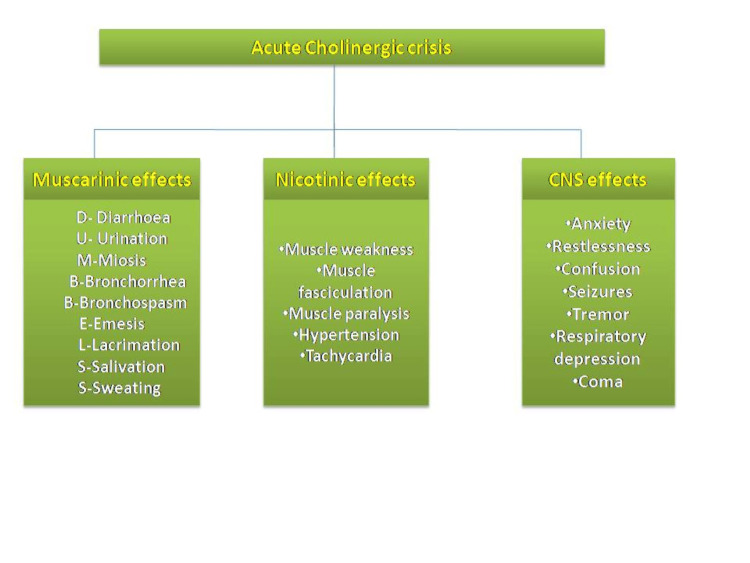
Clinical features of an acute cholinergic crisis Image credits: Akankshi Oberoi and Jayksh Chhabra

The neurological effects seen in OP poisoning can be categorized into three groups, i.e., type 1 paralysis and acute cholinergic crisis, which are often seen at the time of presentation to the hospital. Clinical features are illustrated in Figure 2. 

**Figure 3:**
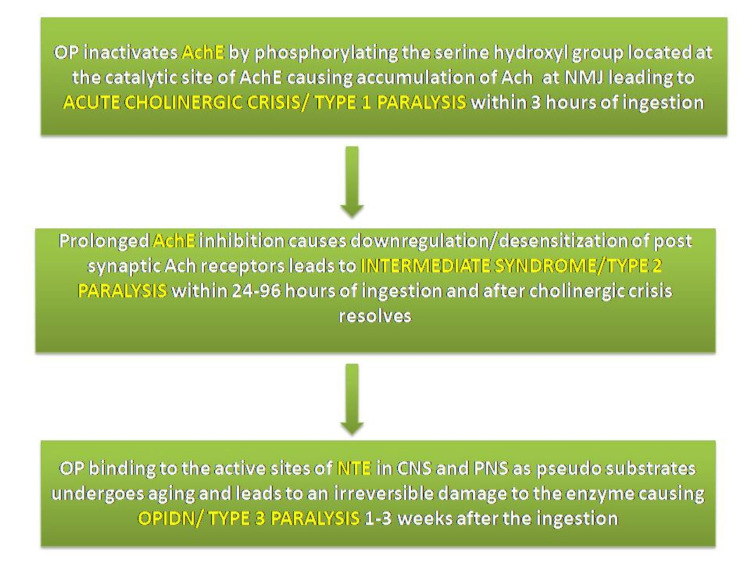
Mechanism of stages of OP poisoning Ach, acetylcholine; AchE, acetylcholine esterase; NTE, neuropathy target esterase; OP, organophosphates; OPIDN, organophosphate-induced delayed neurotoxicity Image credits: Akankshi Oberoi and Jayksh Chhabra

Type 2 paralysis/intermediate syndrome usually begins after 24 hours of ingestion and is characterized by paralysis of the neck flexors, respiratory muscles, and limb muscles [5]. OPIDN, which is type 3 paralysis, is characterized by delayed ataxia and Wallerian-type degeneration of the axon and myelin in the most distal regions of the longest tracts in both central and peripheral nervous system pathways [6]. It usually takes one to three weeks to develop [7]. Figure 3 illustrates the pathogenesis of different stages of paralysis after OP ingestion.

OPIDN presents in a series of phases: initially, it is dormant, i.e., “latent phase,” then advancing steadily, i.e., “progressive phase,” reaching a plateau, i.e., “stationary phase,” and finally, showing signs of improvement, i.e., “improvement phase” [6]. The latent period refers to the time interval until neurological symptoms appear. Motor-sensory polyneuropathy develops quickly in the progressive phase. Our patient initially presented in a progressive phase after five weeks of ingestion with bilateral foot drop, sensory impairment in bilateral distal lower limbs, and weakness in the lower limbs. Neurological symptoms continue in the stationary phase, and sensory symptoms resolve in the improvement phase before motor symptoms. This also implies a swift regeneration of the peripheral nerves, therefore unmasking the central lesions of the spinal cord, as seen in our case, which presented later with bilateral lower limb spasticity [8]. Our case shows clinically evaluated myelopathy, given the normal MRI spine. Myelopathy with normal MRI has been reported by Porey et al. [9]. Neuropathy target esterase (NTE) is found in the neuronal endoplasmic reticulum and is involved in transporting materials from the neuronal cell body to axons and dendrites, as well as in membrane trafficking and microtubule-based transport. This function is crucial for maintaining axons. When this maintenance is disrupted, it can lead to significantly swollen axons in the longest nerves of the legs and spinal cord, resulting in delayed neurotoxicity. It leads to functional impairment of distal segments of sensory and motor axons of peripheral nerves, as well as impacting the ascending and descending spinal tracts [10]. Pyramidal tract signs as a sign of CNS damage, which are usually observed in the latter part of the disease, were also discussed by Senanayake [11].

**Figure 4:**
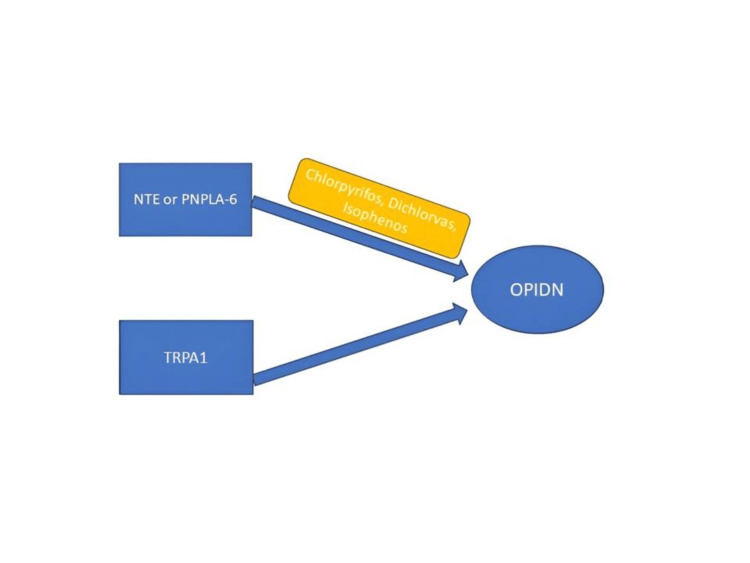
Mechanism of OPIDN NTE, neuropathy target esterase; OPIDN, organophosphate-induced delayed neurotoxicity; PNPLA-6, patatin-like phospholipase domain-containing protein 6; TRPA1, transient receptor potential cation channel member A1 Image credits: Jayksh Chhabra and Akankshi Oberoi

NTE, also known as patatin-like phospholipase domain-containing protein 6 on chromosome 19p13.2, plays a key role in OPIDN [12]. Although the exact mechanism of the NTE in causing the OPIDN is unknown, Glynn explained that OP compounds bind to the active sites of the NTE as if they were substrates, but unlike real substrates, their hydrolysis rate is prolonged. Additionally, these compounds undergo a unique secondary process known as aging, which is specific to them. During aging, one of the attached alkyl groups of the phosphoryl residue is cleaved, creating a negatively charged phosphate at the enzyme’s active site, resulting in irreversible damage to the enzyme [13]. After a delay of one to three weeks, these events result in neuropathy, characterized by the degeneration of long axons in neurons. Furthermore, a recent study has revealed a more modern method that involves the activation of transient receptor potential cation channel member A1 (TRPA1) by OP, which eventually leads to myelin destruction, as illustrated in Figure 4.

TRPA1 plays a crucial role in developing OPIDN, and inhibiting TRPA1 is a productive approach for treating OPIDN, as discussed by Ding et al. [14]. Sahoo et al. assessed three cases of OPIDN using the Overall Neuropathy Limitation Scale (ONLS) to measure daily activity limitations in the upper and lower limbs. They recorded ONLS scores at admission and follow-ups. The results showed significant improvement, with the upper limbs nearly returning to normal and some residual deficits in the lower limbs, which could improve over time. These results were seen at follow-up after two years, three years, and seven months, respectively, for three cases, therefore demonstrating that rehabilitative therapy and good nursing care can improve the effects of neuropathy [15].

## Conclusions

OP-induced clinically evaluated myelopathy with a normal MRI highlights the importance of thorough clinical assessment in patients with neurological deficits. Clinicians should be highly vigilant for OPIDN in patients presenting with neurological symptoms after exposure to OP, even if MRI results are normal. Early recognition and symptomatic treatment can help improve the quality of life of the patients. Further research is essential to better understand the condition and improve diagnostic and treatment approaches.
